# Six macrophage-associated genes in synovium constitute a novel diagnostic signature for osteoarthritis

**DOI:** 10.3389/fimmu.2022.936606

**Published:** 2022-07-28

**Authors:** Yiying Liu, Taoyuan Lu, Zaoqu Liu, Wenhua Ning, Siying Li, Yanru Chen, Xiaoyong Ge, Chunguang Guo, Youyang Zheng, Xiangyang Wei, Haiming Wang

**Affiliations:** ^1^ Academy of Medical Sciences, Zhengzhou University, Zhengzhou, China; ^2^ Department of Rehabilitation Medicine, The First Affiliated Hospital of Zhengzhou University, Zhengzhou, China; ^3^ Department of Cerebrovascular Disease, Zhengzhou University People’s Hospital, Henan Provincial People’s Hospital, Zhengzhou, China; ^4^ Department of Interventional Radiology, The First Affiliated Hospital of Zhengzhou University, Zhengzhou, Henan, China; ^5^ Department of Endovascular Surgery, The First Affiliated Hospital of Zhengzhou University, Zhengzhou, China; ^6^ Department of Cardiovascular Medicine, The First Affiliated Hospital of Zhengzhou University, Zhengzhou, China; ^7^ Medical College of Zhengzhou University of Industrial technology, Zhengzhou, China

**Keywords:** Osteoarthritis, synovial macrophages, macrophage-associated genes, machine learning, diagnostic signature, immunopathology

## Abstract

**Background:**

Synovial macrophages play important roles in the formation and progression of osteoarthritis (OA). This study aimed to explore the biological and clinical significance of macrophage-associated genes (MAGs) in OA.

**Methods:**

The OA synovial gene expression profiles GSE89408 and GSE82107 were obtained from the GEO database. Single-sample gene set enrichment analysis (ssGSEA) and GSEA were employed to decipher differences in immune infiltration and macrophage-associated biological pathways, respectively. Protein–protein interaction (PPI) network analysis and machine learning were utilized to establish a macrophage-associated gene diagnostic signature (MAGDS). RT-qPCR was performed to test the expression of key MAGs in murine models.

**Results:**

OA synovium presented high levels of immune infiltration and activation of macrophage-associated biological pathways. A total of 55 differentially expressed MAGs were identified. Using PPI analysis and machine learning, a MAGDS consisting of IL1B, C5AR1, FCGR2B, IL10, IL6, and TYROBP was established for OA diagnosis (AUC = 0.910) and molecular pathological evaluation. Patients with high MAGDS scores may possess higher levels of immune infiltration and expression of matrix metalloproteinases (MMPs), implying poor biological alterations. The diagnostic value of MAGDS was also validated in an external cohort (AUC = 0.886). The expression of key MAGs was validated in a murine model using RT-qPCR. Additionally, a competitive endogenous RNA network was constructed to reveal the potential posttranscriptional regulatory mechanisms.

**Conclusions:**

We developed and validated a MAGDS model with the ability to accurately diagnose and characterize biological alterations in OA. The six key MAGs may also be latent targets for immunoregulatory therapy.

## Introduction

Osteoarthritis (OA) is a degenerative and chronic joint disease characterized by clinical symptoms and distortion of joint tissues (1), affecting an estimated more than 240 million people worldwide (2). Multiple factors, mainly mechanical injury and immune disorder, mediate a series of complex pathological changes including cartilage degradation, synovial inflammation, subchondral bone remodeling, and osteophyte formation, which synergistically drive the formation and progression of OA (3). Immunological changes such as chronic inflammation play a central role in these pathological processes, of which synovial macrophages are considered to be crucial immune activation mediators (4, 5). Hence, it is extremely valuable to systematically investigate synovial macrophage-associated biological alterations, which can provide a reference for an in-depth understanding of the immunopathological mechanisms and the development of novel molecular diagnostic techniques in OA.

The synovium is the main diseased tissue in OA due to its pathological inflammation. Synovial inflammation mediates cartilage and subchondral bone-related pathological changes by promoting the expression of various matrix-degrading enzymes, resulting in joint pain and dysfunction (6). Immune cells are the main mediators of synovial inflammation. Accumulated immune cells such as macrophages (7) and immune-related molecules such as interleukins (8) and chemokines (9) are considered to be major mediators. In recent years, synovial macrophages are considered essential injury-driving immune cells in OAs because of their multifaceted roles such as immune recruitment, inflammatory injury, and mediating extracellular matrix (ECM) degradation (10). Activated macrophages can polarize into M1- and M2-like macrophages, playing proinflammatory and anti-inflammatory roles in OA, respectively (11). Under pathological conditions, the balance of proinflammatory and anti-inflammatory driven by M1/M2 macrophages is broken, resulting in immunogenic damage to osteoarticular tissues and contributing to the resting pain symptom (12). In addition, accumulation of synovial macrophages also contributes to osteoarthritis joint pain through various mechanisms, such as pain sensitization (13), high expression of the neurotrophin nerve growth factor (14), and neuroimmune cross talk (15). Taken together, synovial macrophages are important mediators of immune activation and related pathologies in OA, but their transcriptional changes associated with macrophage activation have not been fully clarified.

OA is a degenerative disease that is difficult to reverse, with late manifestations of joint pain, deformity, and other symptoms, which seriously affect activities of daily living. Therefore, early diagnosis and timely intervention are extremely important, as they help to delay the course of the disease and relieve symptoms. In recent years, an increasing number of studies have confirmed that various dysregulated genes, proteins, and other molecules in OA can be used as important diagnostic markers and therapeutic targets. C-terminal cross-linked telopeptides of type II collagen (CTX-II) are one of the most frequently assessed markers for OA diagnosis (16). Synovial fluid peptidase activity could play a role as a measure of disease burden and a predictive biomarker of progression in knee OA (17). Interstitial stem cells have the potential for the clinical treatment of OA due to their effective immunomodulatory properties (18). Considering the key roles of macrophages in immune activation and related pathological changes in OA, exploring the biological and clinical significance of macrophage-associated genes (MAGs) may also contribute to advances in OA molecular diagnosis and immunomodulatory therapy.

In the present study, we performed a systematic investigation of MAGs in OA synovium, aiming to identify key MAGs and construct a multigene signature for OA molecular diagnosis. Single-sample gene set enrichment analysis (ssGSEA) and GSEA revealed differences in immune infiltration and macrophage-associated biological pathways between OA and normal synovium. A macrophage-associated gene diagnostic signature (MAGDS) for auxiliary diagnosis of OA was established using protein–protein interaction (PPI) network analysis and machine learning, and the potential biological significance underlying MAGDS was also explored. In addition, a competitive endogenous RNA (ceRNA) network targeting six key MAGs was constructed to provide a reference for understanding of the posttranscriptional regulatory mechanism and molecular therapy options in OA.

## Materials and methods

### Public data collection and processing

**Figure 1:**
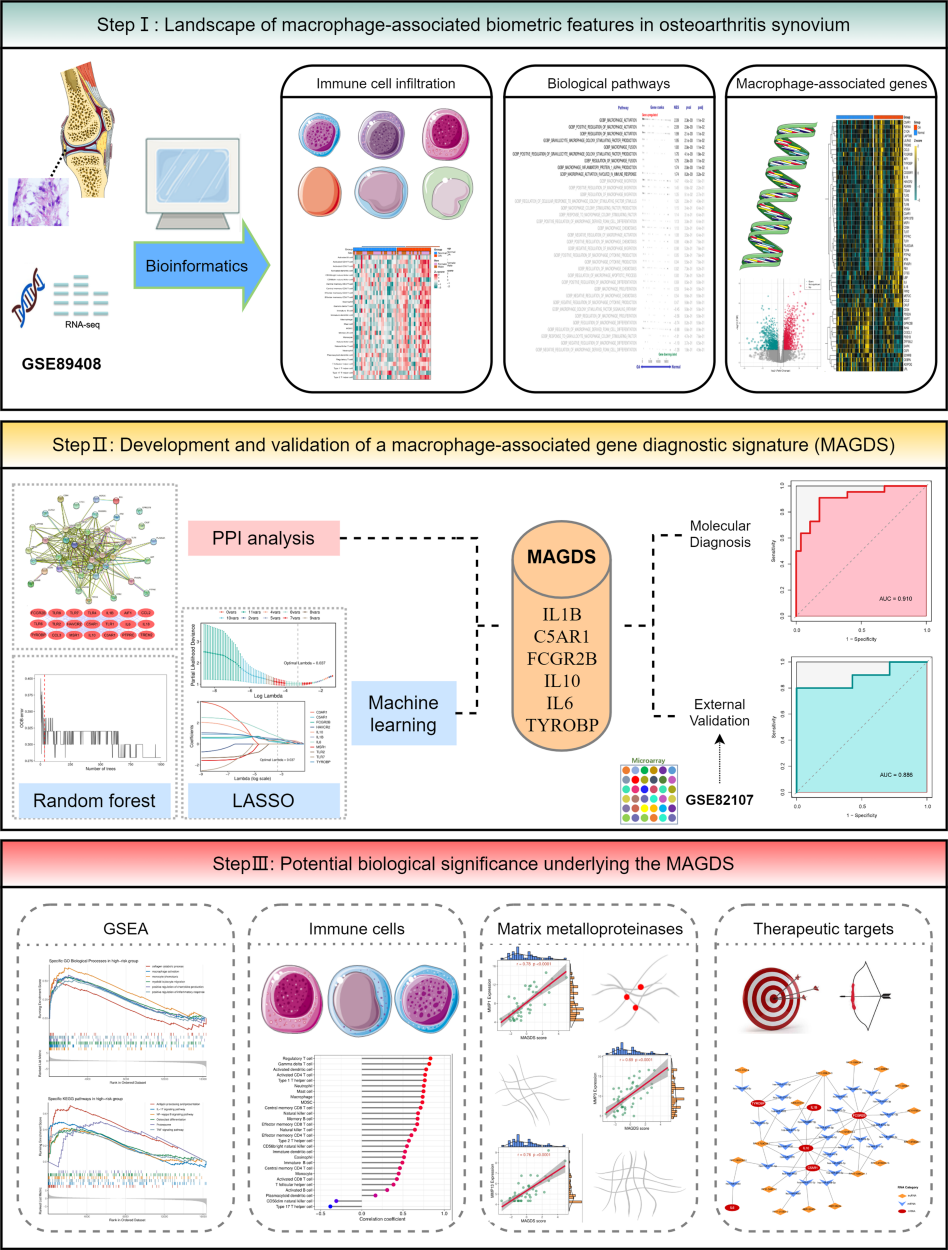
The overall workflow of this study.

The overall workflow of this study is depicted in **Figure 1**. In this study, two OA synovial gene expression profiles were recruited from the Gene Expression Omnibus (GEO, http://www.ncbi.nlm.nih.gov/geo/) database, including a high-throughput RNA sequencing dataset GSE89408 and a microarray dataset GSE82107. The detailed baseline information is summarized in **Supplementary Table S1**. GSE89408, containing 22 OA synovium samples and 28 normal controls, was applied to investigate macrophage-associated biological characteristics and construct a diagnostic signature in OA, and GSE82107 containing 10 OA synovium samples and 7 normal controls was employed for external validation of the diagnostic signature. RNA read count data were normalized to variance-stable transformed (VST) values using the DESeq2 R package. The microarray data were preprocessed *via* quantile normalization and log2 transformation *via* the limma R package. The GENCODE database (https://www.gencodegenes.org/) was applied for mRNA and long non-coding RNA (lncRNA) annotations.

### Immune cell infiltration estimation

The gene set for marking 28 immune cell types was enrolled from a previously published article (19) and illustrated in **Supplementary Table S2**. Then, the relative infiltration abundance of immune cells in OA and normal synovium was estimated by the ssGSEA algorithm implemented in the GSVA R package, which is broadly utilized in immune infiltration-related bioinformatics studies (20–23).

### Macrophage-associated gene set collection

To explore the macrophage-associated biological characteristics in OA, we searched the Molecular Signatures Database (MSigDB, http://www.gseamsigdb.org/gsea/msigdb/index.jsp) with “macrophage” as the keyword and C5–Gene Ontology (GO) as the filtering condition, which returned 35 macrophage-associated gene sets containing a total of 240 MAGs. Please refer to **Supplementary Table S3** for details of the 35 gene sets.

### Gene set enrichment analysis

GSEA was performed on the 35 macrophage-associated gene sets *via* the clusterProfiler R package to identify markedly aberrant macrophage-associated biological alterations in OA. Permutations were set to 10,000 to obtain normalized enrichment scores (NESs) in GSEA. Gene sets with adjusted P value <0.05 were considered significantly enriched. The fgsea R package was employed to display the enrichment results. GSEA was also utilized to explore potential biometric differences between high- and low-risk groups distinguished by signature scores.

### Identification and functional enrichment of differentially expressed MAGs

In GSE89408, differentially expressed genes (DEGs) between OA and normal conditions were identified by criteria of absolute value of log2FoldChange greater than 0.5 and a false discovery rate (FDR) less than 0.05. MAGs overlapping with the DEGs were defined as DEMAGs in OA. Furthermore, the clusterProfiler R package was applied to perform GO and Kyoto Encyclopedia of Genes and Genomes (KEGG) enrichment analyses of the DEMAGs. Terms and pathways with adjusted P value <0.05 were considered significantly enriched.

### PPI network analysis of DEMAGs

The PPI network of the DEMAGs was constructed *via* the STRING database (https://www.string-db.org/). The critical subnetwork and hub genes in the PPI network were further identified utilizing the MCODE plug-in of Cytoscape software.

### Establishment and validation of the MAGDS model

Prior to constructing the MAGDS model, we transformed gene expression data into z-score in both GSE89408 and GSE82107, which enhanced the comparability between the different datasets. The machine learning-based development procedure for the MAGDS model in the training set GSE89408 was as follows (1): The random forest algorithm was employed to screen important gene variables that could distinguish OA from control conditions, with a filter condition of relative importance greater than 0.5. (2) These random forest-screened important genes and the hub genes identified in the PPI network were intersected, and the resulting genes were selected as candidate modeling genes. (3) Ultimately, least absolute shrinkage and selection operator (LASSO) regression was applied to construct the MAGDS model. The diagnostic value of the MAGDS and model genes was estimated *via* receiver operating characteristic (ROC) curve analysis. We then introduced the GSE82107 dataset as an external validation cohort to test the diagnostic power and robustness of the model.

### Construction of ceRNA networks

The ceRNA regulatory network of model genes was constructed through the following pipelines: First, differentially expressed lncRNAs (DElncRNAs) annotated by the GENCODE database were extracted from all DEGs and included in the ceRNA network analysis. Second, target microRNAs (miRNAs) of these DElncRNAs were predicted according to lncBase v.2 with a threshold of miTG score >0.99. Third, miRNAs targeting the six vital DEMAGs were predicted *via* miRWalk (http://mirwalk.umm.uni-heidelberg.de/), filtering with miRWalk Score =1. Fourth, the miRNA–mRNA and lncRNA–miRNA binding pairs obtained from the above steps were merged into multiple lncRNA–miRNA–mRNA regulatory axes. Only regulatory axes with significant positive correlations between lncRNAs and mRNAs were retained, constituting the final ceRNA network of the six vital DEMAGs. In addition, the ceRNA network was visualized in Cytoscape software.

### OA murine model

The animal study was reviewed and approved by the Ethics Committee for Research and Clinical Trials of the First Affiliated Hospital of Zhengzhou University. The destabilization of the medial meniscus (DMM) surgery was performed on the right knee of 8-week-old C57BL/6 male mice to obtain OA murine models, according to the previously reported method (24). Hematoxylin and eosin (HE)-stained sections were exploited to ascertain the gross structure of knee joints in murine models.

### Real-time quantitative polymerase chain reaction

Total RNA was isolated from knee joint synovial tissue of the OA model and normal mice with TRIzol Reagent (Invitrogen, CA, USA) and purified using isopropanol, 75% ethanol, and RNase-free water following the introductions of the manufacturer. After determination of the RNA concentration and purity, cDNA was synthesized using the HiScript^®^ III All-in-one RT SuperMix Perfect (Vazyme Biotech Co., Ltd.). RT-qPCR was conducted using the ChamQ Universal SYBR qPCR Master Mix (Vazyme Biotech Co., Ltd.) by QuantStudio 3 Real-Time PCR System (Thermo Fisher Scientific). The expression level was quantized by 2^-ΔΔCT^ mode. GAPDH was regarded as the reference gene for quantitative analysis. The primer sequences for RT-qPCR are listed in **Supplementary Table S4**.

### Statistical analysis

All data processing, statistical analysis, and plotting were conducted in R 4.1.0 software. Significance was assessed *via* Student’s t test or the Wilcoxon rank-sum test for comparisons of two groups and the Kruskal–Wallis test for comparisons of three or more groups. Correlations between two continuous variables were determined using Pearson correlations. Random forest and LASSO regression were implemented *via* the randomForest and glmnet R packages, respectively. ROC curve analysis was performed *via* the pROC R package. All statistical tests were two-tailed, and P <0.05 was deemed statistically significant.

## Results

### The landscape of immune infiltration in OA synovium

**Figure 2:**
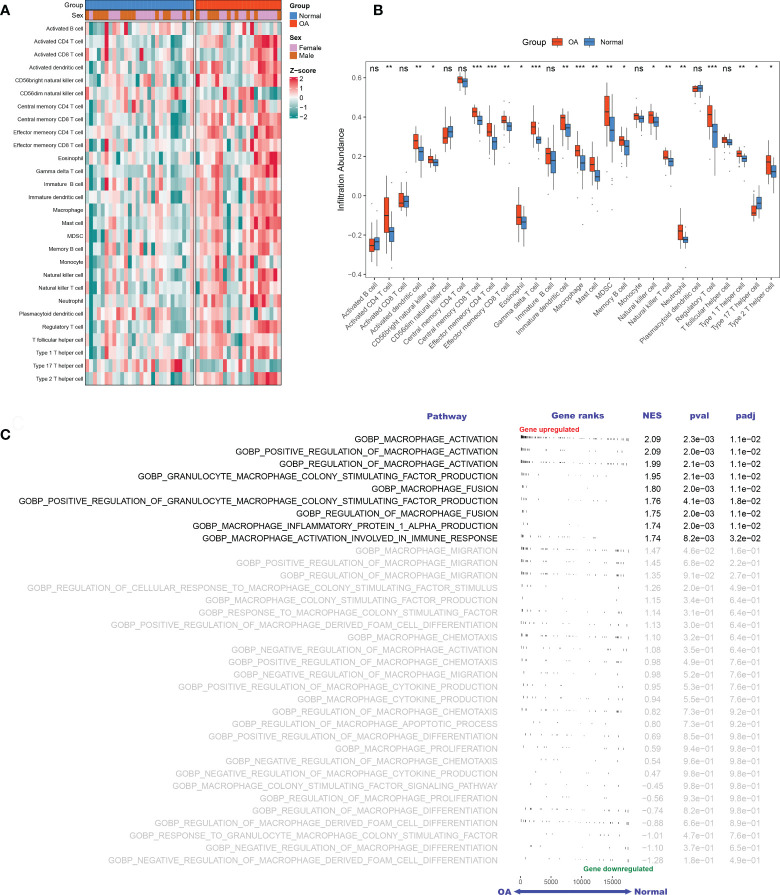
The landscape of immune infiltration and aberrant signaling pathways. A-B. Heatmap **(A)** and comparison boxplot **(B)** of 28-immune-cell infiltration between OA and control groups. ns, (not significant) P > 0.05; *P < 0.05, **P < 0.01, ***P < 0.001. **(C)**. GSEA analysis of 35 macrophage-associated biological pathways between OAs and controls. NES, normalized enrichment score.

Based on the GSE89408 dataset, we applied the ssGSEA method to decode the relative infiltration abundance of 28 immune cell subpopulations in OA synovium and normal controls (**Figure 2A**). Compared with normal controls, OA synovium exhibited a higher infiltration abundance of most immune cells, which suggested a microenvironment of excessive immune activation (**Figure 2B**). Unsurprisingly, the abundance of macrophages was markedly elevated in OA synovium, consistent with previous studies (25). Moreover, innate immune cells, such as mast cells, natural killer (NK) cells, and neutrophils, and adaptive immune cells, such as activated CD4+ T cells, effector memory CD4+ T cells, and Th1 cells, were also significantly increased in the OA synovium, implying that multiple immune cells are involved in the formation of synovial inflammation in OA.

### Identification of DEMAGs

With “Macrophage” as the keyword, we searched the MSigDB database and generated 35 relevant pathways containing 240 MAGs. Based on these biological pathways, we performed GSEA and detected that macrophage activation, macrophage colony factor-stimulating production, macrophage fusion, and macrophage inflammation protein-1 α production were markedly activated in OA (**Figure 2C**), suggesting the extensive activation of synovium macrophages.

**Figure 3:**
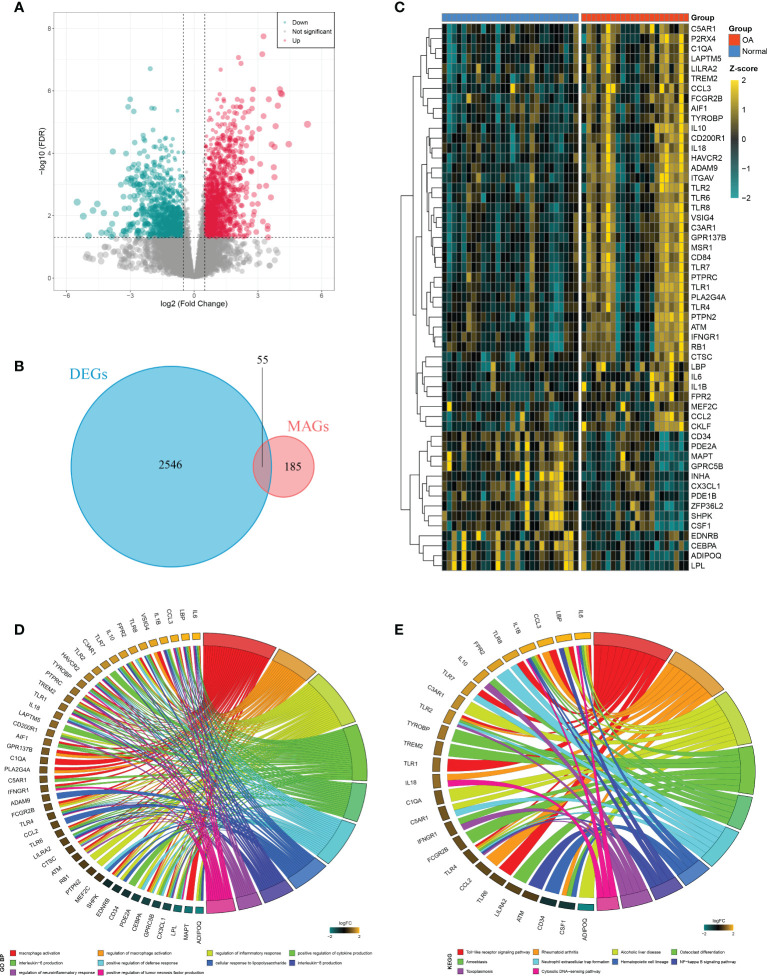
Identification of DEMAGs and functional annotation. **(A)** Volcano plot of DEGs between OAs and controls. **(B)** Venn diagram showed the intersection of MAGs and DEGs. **(C)** Heatmap of the expression of the DEMAGs. D-E. GO **(D)** and KEGG **(E)** enrichment analyses of DEMAGs.

A total of 2,601 DEGs were identified between OAs and normal controls, including 1,527 upregulated genes and 1,074 downregulated genes (**Figure 3A**). Taking the intersection of these DEGs with 240 MAGs, 55 DEMAGs were detected in OA synovium (**Figures 3B, C**). GO and KEGG enrichment analyses indicated that these DEMAGs were mainly enriched in biological processes of macrophage activation, IL6 and IL8 production, regulation of macrophage activation, inflammation, cytokine production, and tumor necrosis factor production, as well as relevant pathways such as the Toll-like receptor and NF-kappa B signaling pathways (**Figures 3D, E**). We thus speculated that these DEMAGs were closely related to the activation and migration of synovial macrophage and were promising biomarkers and therapeutic targets in OA.

### Generation of MAGDS from PPI analysis and machine learning

**Figure 4:**
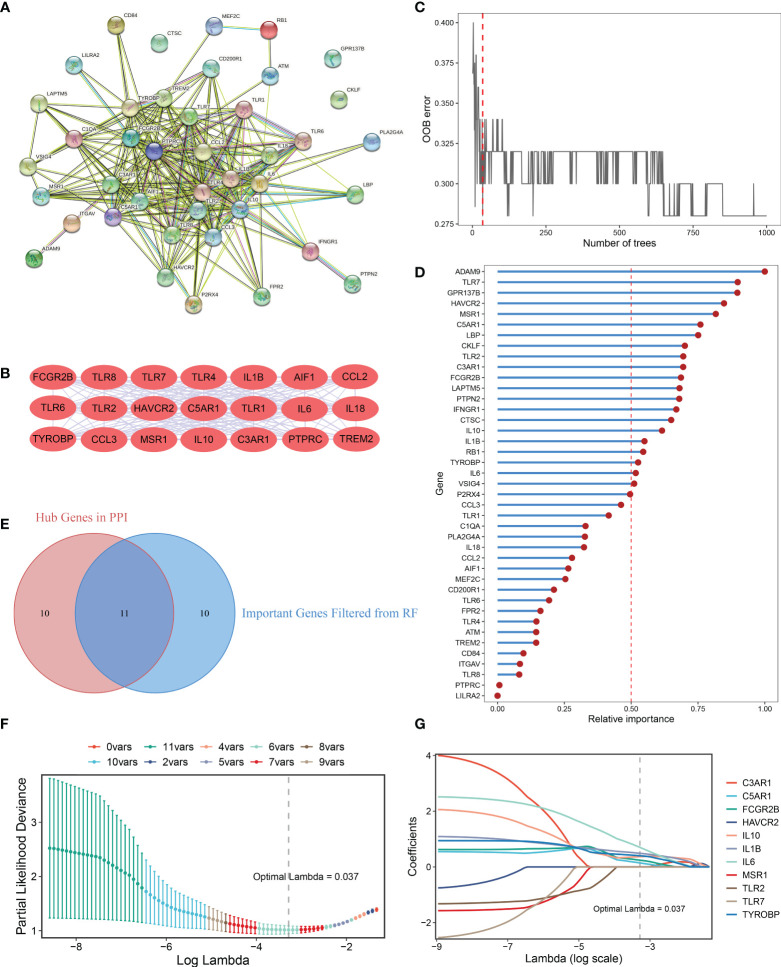
Integrative construction of the MAGDS. **(A)** Protein-Protein Interaction (PPI) network of DEMAGs. **(B)** The 21 red nodes we highlighted in the network were hub genes identified by cytoHubba-degree algorithm. **(C)** Out of bag (OOB) error rate reached a minimum when the number of trees was equal to 35. **(D)** Relative importance of 41 up-regulated DEMAGs calculated in random forest. **(E)** Venn diagram of the intersection of important gene variables obtained from PPI analysis and random forest pre-screening. **(F)** The optimal lambda was determined when the partial likelihood deviance reached the minimum value. **(G)** LASSO coefficient profiles of the candidate genes for MAGDS construction.

A PPI network representing the relationship of proteins encoded by the 41 upregulated DEMAGs was generated using the STRING database (**Figure 4A**). The MCODE plug-in of Cytoscape software was utilized to extract the hub subnetwork from the PPI network, resulting in 21 hub genes (**Figure 4B**). We also employed a random forest algorithm to screen key gene variables capable of distinguishing OA and controls, with a relative importance greater than 0.5 as the filtering criterion. The out-of-bag (OOB) error rate reached a minimum when the number of trees was equal to 35 (**Figure 4C**), and 21 of 41 genes were selected as important variables (**Figure 4D**). By intersecting the hub genes identified in the PPI network and the important genes extracted *via* random forest, we ultimately obtained 11 candidate genes for the final LASSO regression modeling (**Figure 4E**). Based on the fivefold cross-validation, the model reached an optimum when lambda was equal to 0.037, containing six key gene variables (**Figure 4F, G**). Based on the expression of the six model genes, a risk score for each patient was calculated using the following formula: MAGDS score = 0.489 * IL1B + 0.144 * C5AR1 + 0.242 * FCGR2B + 0.369 * IL10 + 0.702 * IL6 + 0.400 * TYROBP - 0.207.

### Diagnostic value of MAGDS

**Figure 5:**
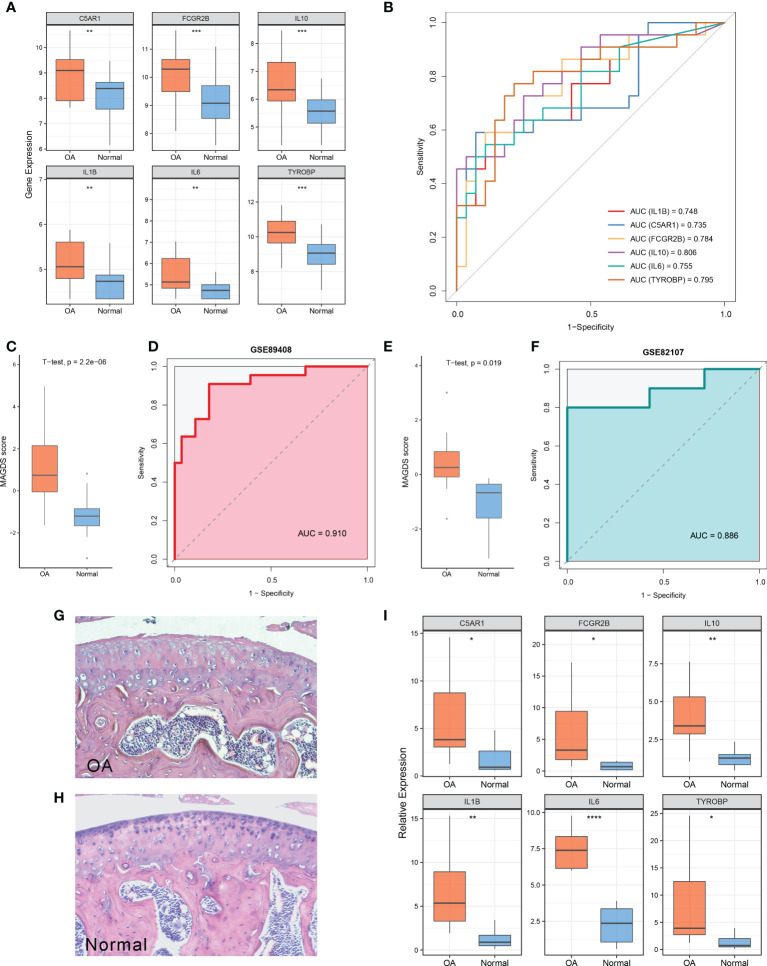
Diagnostic and predictive values of MAGDS. **(A)** Differential expression of the six model MAGs between OA and normal groups. **(B)** ROC analysis of the six key MAGs for OA diagnosis. **(C)** The distribution of MAGDS scores between OAs and controls in GSE89408. **(D)** ROC analysis of MAGDS for OA diagnosis in GSE89408. **(E)** The distribution of MAGDS scores between OAs and controls in GSE82107. **(F)** ROC analysis of MAGDS for OA diagnosis in GSE82107. **(G, H)**. HE staining of paraffin sections of knee joints from OA **(G)** and normal mice **(H)**. Magnification, ×100. **(I)** Relative expression of the six model MAGs between OA and normal mice. Statistic tests: two-sided t-test. ns P > 0.05, *P < 0.05, **P < 0.01, ***P < 0.001, and ****P < 0.0001.

Relative to normal controls, the expression levels of these six model MAGs were significantly higher in OA (**Figure 5A**). The AUCs of IL1B, C5AR1, FCGR2B, IL10, IL6, and TYROBP for OA diagnosis were 0.748, 0.735, 0.784, 0.806, 0.755, and 0.795, respectively (**Figure 5B**), which indicated that these six key MAGs were valuable biomarkers for molecular diagnosis in OA. Furthermore, the diagnostic capability of the MAGDS model was estimated and validated. In the training cohort GSE89408, MAGDS scores were higher in OA patients, and the AUC of MAGDS for OA diagnosis was 0.910, suggesting a superior performance (**Figures 5C, D**). Furthermore, we introduced an external validation cohort GSE82107 to verify the diagnostic power of the signature. In this cohort, MAGDS scores were still higher in the OA group, and its diagnostic AUC was 0.886, indicating the good accuracy and stability of MAGDS for OA diagnosis (**Figures 5E, F**).

### Validation of the expression level of the six key MAGs in a murine model

HE staining showed the histological features of knee joints in OA and normal mice (**Figures 5G, H**). OA mice were characterized by cartilage superficial destruction, cartilage erosion, and obvious hyperplasia of the basal layer. Next, the expression levels of the six key MAGs in synovial tissues of OA mice (n = 10) and healthy controls (n = 8) were measured using RT-qPCR. As shown in **Figure 5I**, C5AR1 (P = 0.022), FCGR2B (P = 0.027), IL10 (P = 0.007), IL1B (P = 0.004), IL6 (P < 0.001), and TYROBP (P = 0.023) were significantly higher in OA samples than those in healthy controls, consistent with the aforementioned findings in human synovial tissues.

### Biological significance underlying MAGDS

**Figure 6:**
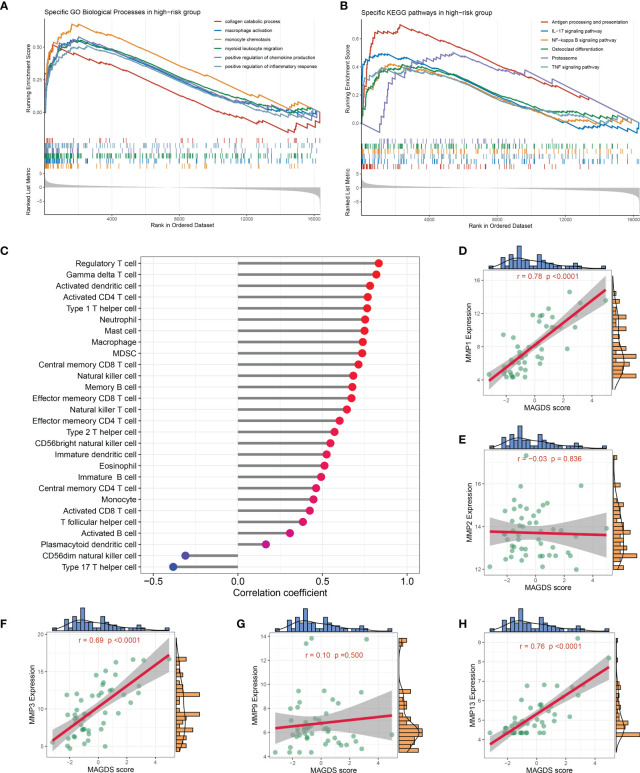
Biological significance underlying MADGS. A-B. GO **(A)** and KEGG **(B)** gene set enrichment analysis performed between high- and low-risk groups. **(C)** Relationship between MAGDS and immune cell abundance. **(D-H)**. Correlation between MAGDS scores and the expression of MMP1 **(D)**, MMP2 **(E)**, MMP3 **(F)**, MMP9 **(G)**, and MMP13 **(H)**.

We separated all samples in GSE89408 into high- and low-risk groups based on the median MAGDS score and performed GSEA to further explore the potential biological mechanisms underlying MAGDS. Numerous pathways associated with immune inflammatory activation and ECM metabolism, such as the collagen catabolic process, macrophage activation, monocyte chemotaxis, myeloid leukocyte migration, positive regulation of the inflammatory response, antigen processing and presentation, osteoclast differentiation, proteasome, IL-17 signaling pathway, NF-kappa B signaling pathway, and TNF signaling pathway, were enriched in the high-risk group (**Figures 6A, B**). These findings suggested that high MAGDS scores may be closely related to immune activation and ECM metabolism, both of which are the major pathological changes mediating cartilage damage and degeneration in OA. Thus, we further explored the correlation between MAGDS and immune cells and MMPs. As shown in **Figure 6C**, MAGDS was positively correlated with most innate and adaptive immune cells, such as regulatory T cells, activated CD4+ T cells, activated dendritic cells, mast cells, neutrophils, and macrophages, but negatively linked to CD56- natural killer cells and type 17 T helper cells, which implied the ability of MAGDS to reflect immune cell infiltration in the synovium of OA patients. According to previous studies, MMP1, MMP2, MMP3, MMP9, and MMP13 are vital ECM metabolism-relevant enzymes in OA. Correlation analysis between MAGDS and these MMPs indicated that MAGDS was positively associated with the expression of MMP1 (r = 0.78, P < 0.0001), MMP3 (r = 0.69, P < 0.0001), and MMP13 (r = 0.76, P < 0.0001) but not with MMP2 (r = -0.03, P = 0.836) or MMP9 (r = 0.10, P = 0.500; **Figures 6D–H**). These results suggested that MAGDS may also have the capacity to reflect ECM metabolism levels to some extent. Taken together, patients with high MAGDS scores may experience a series of adverse biological changes, including high levels of immunoinflammatory activity and ECM hypermetabolism. From a molecular pathological perspective, these patients may be at high risk of tissue damage and disease progression, requiring intensive attention and timely treatment.

### Potential ceRNA network for the six MAGs

**Figure 7:**
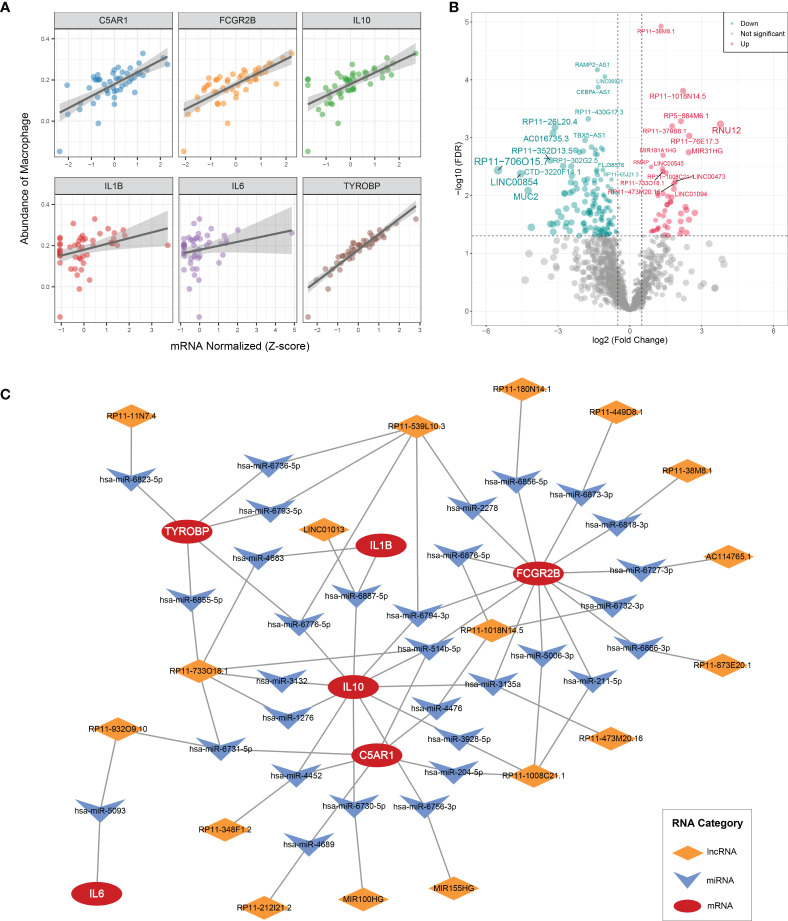
CeRNA regulatory networks **(A)** Correlation analysis of six key MAGs and macrophages. **(B)** Volcano plot of DElncRNAs between OAs and controls. **(C)** CeRNA networks of IL1B, C5AR1, FCGR2B, IL10, IL6, and TYROBP. Log2FC, log2(foldchange).

IL1B, C5AR1, FCGR2B, IL10, IL6, and TYROBP were all prominently correlated with synovium macrophages (**Figure 7A**), which implied that the six key DEMAGs may be latent molecular therapeutic targets in OA. To unveil the potential posttranscriptional regulatory mechanisms of the six key MAGs, we constructed a ceRNA network according to the aforementioned pipeline. In GSE89408, a total of 184 DElncRNAs were identified and included in the subsequent ceRNA network construction process (**Figure 7B**). MiRNAs targeting the six key DEMAGs and these DElncRNAs were predicted according to miRWalk and lncBase v.2, respectively. After integrating the miRNA–mRNA and lncRNA–miRNA binding pairs, a ceRNA network comprising six MAGs, 31 miRNAs, and 17 lncRNAs was completely constructed (**Figure 7C**). In each lncRNA–miRNA–mRNA regulation axis, lncRNAs may enhance the expression of key MAGs by inhibiting the corresponding miRNAs. For example, miR-5093 may be able to inhibit the expression of IL6 and thereby suppress inflammatory activation, and LINC01013 may indirectly upregulate the expression of IL10 by inhibiting miR-6887-5p, thereby enhancing the anti-inflammatory impact. Overall, the ceRNA network provides a reference for the understanding of potential posttranscriptional regulatory mechanisms and the selection of non-coding RNA therapeutic agents for the six key MAGs.

## Discussion

Macrophages play an essential role in the pathological progression of OA and are involved in immune activation, ECM degradation, cartilage destruction (26), and pain formation (27). Therefore, systematic exploration of macrophage-related gene expression profiles and identification of key genes are particularly necessary for an in-depth understanding of the pathological features of OA, which are also conducive to advances in molecular diagnosis and immunomodulatory therapy. In the present study, we first comprehensively revealed multiple macrophage-associated pathological alterations at the levels of cellular infiltration, biological pathways, and gene expression, providing novel insight into the pathological mechanism of OA. Based on the expression profiles of DEMAGs, we developed a MAGDS model composed of six MAGs that possessed accurate diagnostic performance and the potential to characterize biological features in OA synovium. We also constructed a ceRNA network targeting the six key MAGs, which contributes to understanding posttranscriptional regulatory mechanisms and the selection of therapeutic agents.

In this study, we detected those macrophages and most other immune cells were highly infiltrated in the OA synovium. Previous histology-based studies have also demonstrated macrophage enrichment in OA (28, 29). Due to their essential roles in immune recruitment, inflammatory damage, and ECM degradation, macrophages have received much attention and are thought to be important lesion driver immune cells in OAs. Macrophages lose their stable state and are activated through recognition of exogenous pathogen-associated molecular patterns (PAMPs) and endogenous danger-associated molecular patterns (DAMPs) (26, 30). Activated macrophages secrete inflammatory factors, growth factors, and MMPs, induce chondrocyte senescence and apoptosis and osteophyte formation, and reduce the synthesis of key components of the ECM, such as proteoglycans, aggrecan, and type II collagen. To identify macrophage activation-associated pathogenic genes, we overlapped the MAGs and DEGs, resulting in 55 DEMAGs, which may be vital mediators of macrophage activation in OA. GO and KEGG analyses indicated that DEMAG was enriched in immune activation-related pathways, such as the Toll-like receptor and NF-kappa B signaling pathways, along with a positive regulation of cytokine production and IL6 production, which further supported our inference. Overall, we reveal the immune cell infiltration landscape in OA and identify biological processes linked to macrophage activation as well as dysregulated MAG, providing new insights into the pathogenic mechanisms of synovial macrophage activation in OA.

Using PPI analysis and machine learning, IL1B, C5AR1, FCGR2B, IL10, IL6, and TYROBP were identified as key MAGs in OA. The advantage of the combined application of PPI analysis and machine learning is that it can synthesize the dual screening results from biological and mathematical methods, resulting in key genes with both important biological and diagnostic roles. According to the PPI analysis result and previous reports, these six key MAGs interact with each other and are closely related to immune responses. IL1B and IL6 are important proinflammatory cytokines that are produced by activated macrophages and are critical mediators of the disturbed processes implicated in OA pathophysiology. Elevated levels of IL1 and IL6 have been found in the synovial fluid and synovial membranes of OA patients, confirming their important roles in OA pathogenesis (31). IL10 is a well-known anti-inflammatory and immunoregulatory cytokine produced primarily by immune system cells such as monocytes, macrophages, T cells, NK cells, and B cells, and acts on them. It has been shown that increased IL10 expression is associated with synovial hyperplasia, consistent with macrophage recruitment and activation after injury. Secretion of pro- and anti-inflammatory mediators increases proportionally and almost simultaneously after macrophage activation (32). In summary, IL1B, IL6, and IL10 regulate OA development through inflammatory regulation.

TYROBP, also known as DNAX-activating protein of 12 kDa (DAP12), is a membrane-encoded immune-related signal transduction adaptin that functions *via* a tyrosine-based immunoreceptor activation motif (33). TYROBP is a critical regulator in the immune system and is involved in the proliferation, survival, differentiation, and polarization of immune cells, particularly the monocyte-macrophage system (34, 35). For example, DAP12 expression in lung macrophages regulates neutrophil extravasation and thereby mediates acute non-infectious tissue injury (36). TREM1 enhances the release of proinflammatory cytokines and chemokines in hypoxic mature dendritic cells (mDCs) by activating DAP12-related signaling pathways (37). Therefore, we hypothesize that TYROBP mediates the pathological changes of OA through proinflammatory mechanisms.

C5AR1 is a receptor for the anaphylatoxin C5a expressed on immune cells and bone cells, a powerful chemical attractant and potent inflammatory mediator that elicits and amplifies inflammatory responses by inducing degranulation, cytokine release, and oxidative burst in immune cells after binding to its G-protein-coupled membrane-bound receptor C5AR1 (38). C5a induces the release of proinflammatory cytokines from activated synoviocytes. The interaction between C5a and C5aR induces the release of TNF-α in activated synoviocytes, suggesting that C5AR1 may play an important role in the joint inflammation process (39).

FCGR2B, an inhibitory receptor that is found on most types of immune cells, is a negative regulator of antibody production and inflammatory reactions (40). This genetic variant has long been associated with susceptibility to autoimmune diseases. During the immune response, antigen–IgG immune complexes bind simultaneously with B-cell receptors and FCGR2B to inhibit the biological response of B cells. FCGR2B also downregulates the function of monocytes and macrophages (41). Thus, we hypothesize that FCGR2B may be involved in OA pathogenesis and progression by regulating the functions of B cells, monocytes, and macrophages.

Early diagnosis of OA is important for the management of this disease, as it helps to open a “treatment window” for early interventions, thereby positively modifying the disease process (42). The current diagnosis of OA is based on radiographic changes, which lack a response to the biological status of OA. Molecular markers can be indicative of biological changes. Changes in the biomarker profile detected in synovial fluid may play an important role in the early detection of OA (43), which may have a significant impact on the field of precision medicine in OA.

The analysis of diagnostic value indicated that our MAGDS model possessed high accuracy and stability for OA diagnosis in the training and validation cohorts, which implied great potential for clinical translation of the MAGDS. Notably, MAGDS showed higher accuracy than six candidate biomarker genes alone, consistent with the multimolecular driving nature of OA. In addition, a high MAGDS score represented a higher abundance of immune cell infiltration and ECM metabolism levels, which suggested that MAGDS can serve as an indicator of deleterious molecular pathological alterations in OA patients. Overall, subjects with high MAGDS scores may possess adverse biological alterations, requiring more vigilant attention or prompt therapeutic intervention.

With great advancements in drug delivery technology, molecular targeted therapy appears to be a potential treatment for OA early intervention (44, 45). TissueGene-C has been found to deliver TGF-β1 to target cells, promote the formation and enhance the activity of M2 macrophages in the joints of OA patients, and improve OA symptoms (46, 47). In this study, IL1B, C5AR1, FCGR2B, IL10, IL6, and TYROBP were identified as vital MAGs associated with immunological recruitment, regulation, and macrophage activation in OA, suggesting that they could be exploited as immunoregulatory targets. Of note, our findings also show that all six genes are strongly positively associated with macrophage infiltration abundance, also implying their immune-recruiting or immunomodulatory functions. Diacerein is a drug used to treat OA by interfering with IL1 (48). Systemic blockade of IL6 by MR16-1 alleviated DMM-induced OA cartilage lesions and the degree of synovitis (49). In recent years, non-coding RNAs, notably lncRNAs and miRNAs, have been recognized as key gene expression modulators. MiR-93 inhibits chondrocyte apoptosis and inflammation in osteoarthritis (50). The lncRNA BLACAT1 regulates the differentiation of bone marrow stromal stem cells by targeting miR-142-5p in osteoarthritis (51). A potential ceRNA regulatory network targeting these six genes was constructed in our study, providing a new reference for mechanistic understanding and targeted therapy. For proinflammatory genes, the use of corresponding miRNA preparations may inhibit their expression and then reduce the synovial inflammatory response; for anti-inflammatory genes, such as IL10, the use of corresponding lncRNA preparations or the IL6 inhibitors olokizumab and siltuximab enhances immunosuppressive regulation.

Although our MAGDS exhibited accurate diagnostic power and was validated with an external dataset, some limitations still need to be clearly elucidated. Firstly, the establishment and validation of the MAGDS model were performed on public datasets with small samples; a large-sample validation and optimal cutoff determination are required before clinical translation. Secondly, the recruitment, activation, and regulatory roles of these six genes in macrophages as well as the ceRNA regulatory network require validation in further *in vitro* and *in vivo* experiments.

In conclusion, our study developed and validated a novel macrophage-associated gene signature with the ability to accurately diagnose OA and characterize biological alterations in OA synovium, which may be a promising indicator for assisting clinical decision-making. The six key MAGs, namely, IL1B, C5AR1, FCGR2B, IL10, IL6, and TYROBP, were potential targets for immunoregulatory therapy.

## Data availability statement

The datasets presented in this study can be found in online repositories. The names of the repository/repositories and accession number(s) can be found in the article/**Supplementary Material**.

## Ethics statement

The animal study was reviewed and approved by the Ethics Committee for Research and Clinical Trials of the First Affiliated Hospital of Zhengzhou University.

## Authors contributions

YL, TL, and HW designed this work. YL, TL, and ZL integrated and analyzed the data. YL and TL wrote this manuscript. XW, HW, WN, SL, YC, XG, CG, YZ, and ZL edited and revised the manuscript. All authors contributed to the article and approved the submitted version.

## Funding

This study was supported by the Science and Technology Research Projects of Henan Province (Grant No. 192102310121); Joint Project of Health Committee of Henan Province (Grant No. LHGJ 20190252); Key Project of Henan Provincial Education Department (Grant No. 21A320041); Henan Youth Natural Science Foundation Project (Grant No. 202300410388); and Henan Medical Education Research Project (Grant No. Wjlx2020529).

## Conflict of interest

The authors declare that the research was conducted in the absence of any commercial or financial relationships that could be construed as a potential conflict of interest.

## Publisher’s note

All claims expressed in this article are solely those of the authors and do not necessarily represent those of their affiliated organizations, or those of the publisher, the editors and the reviewers. Any product that may be evaluated in this article, or claim that may be made by its manufacturer, is not guaranteed or endorsed by the publisher.
